# Consumption of cranberry polyphenols enhances human γδ-T cell proliferation and reduces the number of symptoms associated with colds and influenza: a randomized, placebo-controlled intervention study

**DOI:** 10.1186/1475-2891-12-161

**Published:** 2013-12-13

**Authors:** Meri P Nantz, Cheryl A Rowe, Catherine Muller, Rebecca Creasy, James Colee, Christina Khoo, Susan S Percival

**Affiliations:** 1 Department of Food Science & Human Nutrition, University of Florida, Box 110370, Gainesville, FL 32611, USA; 2 Department of Statistics, University of Florida, Gainesville, FL 32611, USA; 3 Ocean Spray Cranberries, Inc., Lakeville, MA 02349, USA

**Keywords:** Cranberry, Proanthocyanidins, Immunity, γδ-T cell, NK cell

## Abstract

**Background:**

Our main objective was to evaluate the ability of cranberry phytochemicals to modify immunity, specifically γδ-T cell proliferation, after daily consumption of a cranberry beverage, and its effect on health outcomes related to cold and influenza symptoms.

**Methods:**

The study was a randomized, double-blind, placebo-controlled, parallel intervention. Subjects drank a low calorie cranberry beverage (450 ml) made with a juice-derived, powdered cranberry fraction (*n* = 22) or a placebo beverage (*n* = 23), daily, for 10 wk. PBMC were cultured for six days with autologous serum and PHA-L stimulation. Cold and influenza symptoms were self-reported.

**Results:**

The proliferation index of γδ-T cells in culture was almost five times higher after 10 wk of cranberry beverage consumption (*p* <0.001). In the cranberry beverage group, the incidence of illness was not reduced, however significantly fewer symptoms of illness were reported (*p* = 0.031).

**Conclusions:**

Consumption of the cranberry beverage modified the *ex vivo* proliferation of γδ-T cells. As these cells are located in the epithelium and serve as a first line of defense, improving their function may be related to reducing the number of symptoms associated with a cold and flu.

**Trial registration:**

ClinicalTrials.gov Identifier: NCT01398150.

## Introduction

Cranberries and cranberry juice are associated with promoting urinary tract health [1,2] However, although the responsibility of immune cells is to survey their environment and prevent bacterial and viral infections from overwhelming the body, much of the literature regarding cranberry research has focused on adherence of bacteria [3-6] rather than modification of immune function.

Four studies have shown the effects of cranberry on immune function in diverse ways: in a rabbit model of infection-induced oxidative renal damage, cranberry reduced inflammation [7]; consumption of a cranberry beverage in a human intervention study resulted in a reduction in pathogen in 42% of the subjects without altering normal vaginal microbiota [6]; lower levels of urinary IL-6 were found in pregnant women after drinking cranberry juice for at least 3 days [8]; and an enhanced generation of anti-lymphoma antibodies was detected in an immuno-competent mouse model of lymphoma [9]. Recent studies have shown effectiveness of cranberry in reducing reoccurrence of urinary tract infections [10-12]. Recent clinical interventions with cranberry have focused on pyuria and bacteriuria [13,14], but did not examine the influence on systemic immunity. These studies suggest that systemic immunity is modified by the bioactive compounds in cranberries, but have not directly assessed it.

An immune cell particularly suited to surveillance of the genitourinary tract is the γδ-T cell, which is strategically located in the epithelium of both the intestine and the reproductive tract. We have shown in human consumption studies with an encapsulated dried fruit and vegetable juice fraction [15], two compounds derived from tea [16], and Concord grape juice [17], that various phytochemicals modify *ex vivo* γδ-T cell proliferation. Phytochemicals and proanthocyanidins from herbal preparations also interact with γδ-T cells in vitro [18-20]. Cranberry polyphenols and proanthocyanidins, then, would seem to be potential candidates for modifying human immunity.

Our objective was to determine if a polyphenol-containing fraction of cranberry would have immuno-modulating activities in humans. Our primary outcome was proliferation of γδ-T cells *ex vivo*; however, the proliferation of other immune cells was also examined. Our secondary outcome was the evaluation of illness symptoms during the 10-week study.

## Materials and methods

### Subjects

A total of 54 healthy subjects (17 men and 37 women), ranging in age from 21 to 50 years, with a body mass index between 18 and 30 kg/m^2^, were recruited by posted advertisements to participate in a 10-wk, double-blind, randomized, placebo-controlled, parallel trial. The University of Florida Institutional Review Board approved the study protocol, and informed written consent was obtained from each subject. Subjects were required to be generally healthy, and exclusion criteria were: taking immunosuppressive drugs, recent or chronic antibiotics, antioxidant supplements or probiotics, or any flavonoid-containing supplements; lactating or being pregnant or on hormone therapy; being a chronic user of non-steroidal anti-inflammatory drugs; having an ongoing infection or hypertension that required medication; consuming more than 14 alcoholic beverages per week, eating more than 7 fruits and vegetables per day or following a vegetarian or strict vegan diet. Participants were not allowed to begin the study if they were ill at the time of the first blood draw. Participants were in contact with the enrolling research assistant by e-mail and telephone, weekly or more often, throughout the study.

The predefined primary outcome was proliferation of γδ-T cells in *ex vivo* culture. Power analysis, (*α* = 0.05 and power = 0.80), based on data where a difference would be significant if the proliferation of γδ-T cells was double that of the placebo group with an expected standard deviation of 1.74, indicated that 13 subjects would be needed per group to detect a statistical difference. A predefined level of compliance was consumption of 80% of the allotted juice.

### Study design

The study was conducted between March and May of 2009, to coincide with normal cold and influenza season in the Southeast (CDC, http://www.cdc.gov/flu/; accessed October 2011). The CDC weekly report of influenza activity in the state of Florida that year was as follows: March, widespread to regional activity; April, local to sporadic activity; May, regional to local activity.

Subjects arrived to the Food Science and Human Nutrition building, for an initial baseline fasting blood draw (Day 0) and were randomly assigned, by drawing cards from an opaque envelope that were numbered either 246 or 638, to receive the experimental treatment [cranberry beverage (CB)] or a placebo beverage (PB), both provided by Ocean Spray Cranberries, Inc. (Lakeville, MA). Both subjects and investigators were blinded regarding the treatment groups. The CB contained cranberry components from juice, filtered water, sugar, natural flavors, citric acid, and sucralose. The PB was a color-(Red 40 and Blue 1), calorie-, and sweetener-matched beverage without cranberry components. The experimental beverage is not commercially available, but was formulated to contain a level of polyphenols similar to that found in commercially available cranberry juice cocktails. The fraction of cranberry components used, derived from the juice of cranberries, was prepared and analyzed by the manufacturer (Table 1). High-performance liquid chromatography analysis of the fraction used to prepare the beverage was conducted (manuscript submitted).

**Table 1:** **Chemical characterization**^**a**^**of the cranberry treatment and placebo beverages**

	**Cranberry beverage**	**Placebo beverage**
Proanthocyanidins, % dwb^b^	65-77%	0-1%
Sugars, % dwb	0.77-1.12%	0%
Anthocyanins, % dwb	6.8-11.3%	Not detected
Organic acids, % dwb	0.5-0.9%	0.1-0.2
Phenolic acids, % dwb	7.1-7.5%	Not tested
Flavonols, % dwb	6.8-10.0%	Not tested
**Total Solids,% dwb**	**87.0-107.8%**	**0.1-1.2%**
Sucralose (μg/mL)	152	149
Vitamin C	Not detected	Not detected
ORAC^c^ (μM AA/g)	62.1	Not detected
Colorant (Red 40/Blue 1)	None	1%
Brix by refractometry (^o^)	0.25	0.23

^a^Characterization of the beverages was by standard methodology and performed by the manufacturer of the beverages. Values were assessed in the lab of SSP by standard methodology and were computed against an ascorbic acid (AA) standard curve. ^b^dwb, dry weight basis ^c^ORAC, oxygen radical absorbance capacity.

Subjects were given bottles containing 450 ml (15 oz) of beverage and instructed to drink one bottle throughout the day, each day, for 70 days (10 wk). Participants were supplied with more bottles than were needed to complete the study, in case scheduling conflicts delayed the second blood draw. To prevent bottles from being discarded, subjects were instructed to bring back any remaining bottles at the end of the study.

Participants were also given a daily illness log to record any cold and influenza symptoms (listed below) they experienced during the 10-wk experimental period. Each day they had to answer this question: Did you have any illness symptoms today? If they answered yes, then they were asked to check the box with each of the symptoms they had that day.

The primary outcome of this study was defined, prospectively, as physiological changes to γδ-T cell proliferation in *ex vivo* culture. Illness symptoms were considered as secondary outcomes because of the small number of subjects, and because they were self-reported. These outcomes were defined as incidence (number of people reporting an illness per group and the number of illnesses per group), duration (total number of days with at least two symptoms and average number of days per group), and total number of symptoms per group. Symptoms listed in the daily diary were: runny or congested nose, cough, sneezing, fever and/or chills, sore throat, headache, wheezing, and intestinal distress (nausea, vomiting, diarrhea, and/or abdominal cramps). Allergy symptoms were not included in the analysis of symptoms. Subjects were instructed as to the different manifestations of colds, influenza and allergies: *Colds*–symptoms occur one at a time, generally last for 5-7 days, yellow/greenish nasal discharge, may or may not be accompanied by a fever, with slight body aches and pains; *Influenza*–symptoms occur rapidly, last up to 14 days, no nasal discharge, often a high fever and sometimes chills, with severe body aches and pains, including headache; *Allergy*–symptoms occur rapidly and all at once, last as long as the allergy-causing agent is present, clear and watery nasal discharge, not associated with a fever, and no body aches or pains. The record of the medications taken during an illness also helped to distinguish allergy from colds and influenza. Subjects were asked to report if they missed class or work, whether they sought medical treatment, if they were prescribed any medications as a result of seeking treatment, which over-the-counter medications they took, and whether they had a significant decrease in normal activities due to illness symptoms.

At 10 wk, study participants returned for a final blood draw and to complete an exit questionnaire. The exit questionnaire included questions to determine if subjects experienced any side effects from the beverage, their estimate of compliance and if they generally adhered to the inclusion/exclusion criteria. To determine efficacy of blinding, subjects reported whether they thought they had received the CB or the PB and were asked why they believed that. Study compliance was assessed by comparing the number of bottles of beverage returned at the end of the study (primary assessment of compliance) with the number they should have returned.

### Blood collection and peripheral blood mononuclear cell (PBMC) isolation

Blood was obtained from fasting subjects on Day 0 (baseline) and at 10 wk. Fasting required eating no food after midnight of the previous day. Blood was collected into one 10 ml sodium heparin tube for PBMC isolation, and one 10 ml SST™ tube (Vacutainer, Becton Dickinson, Franklin Lakes, NJ) to obtain serum. All tubes were processed within 2 h of blood collection. Serum was removed from SST™ tubes after centrifugation (2,000 × g, 10 min, 4°C) and used as autologous serum in culture media. Aliquots of serum were frozen at-80°C for antioxidant analysis. Whole blood was diluted 1:1 with 0.9% NaCl and layered on a gradient (Lympholyte® H Cell Separation Media, Cedarlane Laboratories Ltd., Burlington, NC) to separate PBMC by centrifugation (800 × g, 20 min, 20°C). The mononuclear cell layer was removed and washed twice with RPMI 1640 (Cellgro, Mediatech, Inc., Manassass, VA) complete medium (100,000 U/L penicillin; 100 mg/L streptomycin; 0.25 mg/L fungizone; 50 mg/L gentamicin; 2 mmol/L L-glutamine; 25 mmol/L HEPES buffer). Cell pellets were resuspended in RPMI 1640 complete medium and counted on a hemocytometer.

### Culture of PBMC for proliferation and cytokine production

On Day 0, 1.0 × 10^6^ PBMC in RPMI 1640 complete medium containing 50 μM 2-ME and 10% autologous serum were seeded into two wells of duplicate 24-well tissue culture plates (Costar, Corning Incorporated, Corning, NY). Phytohemagglutinin (PHA-L from *Phaseolus vulgaris*) at a final concentration of 10 μg/ml, was added to one set of wells on each plate, while the other set was brought to volume with RPMI 1640 complete medium. The plates were incubated at 37°C in a humidified 5% CO_2_ atmosphere. After 24 h, supernatant fluids from one plate were harvested and frozen at-80°C for future cytokine analysis. On Day 3, human recombinant IL-2 (BD Biosciences, San Jose, CA), at a concentration of 1 ng/ml, was added to all wells of the second plate, which was incubated until Day 6 when cells were harvested and processed for flow cytometry.

### Flow cytometry

PBMC were analyzed by flow cytometry on Days 0 and 6 of culture, using cell surface markers for identification. Specific cell population numbers were expressed as a percentage of total cells [B cells, monocytes and natural killer (NK) cells] or as a percentage of the CD3^+^ population (αβ-T cells and γδ-T cells). To determine proliferation changes of the various cell types at both blood draws, the fold change of individual subjects was calculated as the ratio of numbers of cultured cells to uncultured cells (Day 6/Day 0). If no proliferation occurred during that time, the ratio would equal 1.0. Then, the fold change before beverage consumption (baseline) was subtracted from the fold change after consumption (10 wk). Antibodies were obtained from eBioscience, Inc. (San Diego, CA) and used to detect specific cell types, as follows: γδ-T cells (PE-CD3 and FITC-γδ-TCR); αβ-T cells (PE-CD3 and FITC- αβ-TCR); NK cells (PE-CD314 and FITC-CD56); B cells (PE-CD19) and Monocytes (FITC-CD14). After staining, cells were washed and fixed with 1% paraformaldehyde. Data was acquired on a BD Biosciences FACSort flow cytometer with CellQuest Pro software (BD Biosciences, San Jose, CA) within 48 h. FlowJo Analysis Software, version 7.5 (Tree Star, Inc., Ashland, OR) was used for data analysis.

### Cytokine level determination in cell culture supernatant fluids

Levels of cytokines (IFN-γ, IL-1α, IL-1β, IL-13, MIP-1β, and TNF-α) secreted by PBMC in culture for 24 h, were quantified using a Human Cytokine Multiplex Immunoassay kit, according to the manufacturer’s directions (Millipore Corp., Billerica, MA). Standards and controls were provided with the kit. The beads were analyzed on a Luminex® 200 instrument (Luminex Corporation, Austin, Texas) with xPONENT 3.1 software. In addition, levels of IL-17 were determined by ELISA according to manufacturer’s directions (eBioscience, San Diego, CA) with kit standards and controls. The fluids were thawed on ice and used undiluted in the assay. Final absorbance was measured at 450 nm on a SPECTRAmax 340PC plate reader and data analyzed using SOFTmax® Pro 5.2 (Molecular Devices, Sunnyvale, CA). All values are expressed as pg/ml.

### Serum oxygen radical absorbance capacity (ORAC)

The ORAC assay consisted of monitoring the inhibition of decay of fluorescein in the presence of the peroxyl radical generator 2,2′azobis 2-amidinopropane dihydrochloride (AAPH). The rate of fluorescence decay was monitored over time by calculating the area under the fluorescent decay curve and quantified using a standard curve of Trolox (0.312-2.5 μM, Fluka Chemical/Sigma). Antioxidant activity in protein-free sera was determined using the ORAC assay, as previously described [21,22] for a 96-well microplate reader, with some modifications. Briefly, at collection sera were diluted 1:5 in 6% meta-phosphoric acid and frozen at -80°C. After thawing, they were centrifuged to precipitate protein (12,000 × g, 5 min, 4°C) and diluted 1:10 in phosphate buffer. Then, 50 μl of each sample were pipetted into duplicate wells. Serum samples for quality control were prepared in our laboratory and used in each assay plate to determine variability. Plate to plate variability was 7.1%. Fluorescein solution (20 nM) was added to all wells. The plate was mixed for 3 minutes, and then equilibrated for 7 minutes to 37°C in a SPECTRAmax GeminiXPS fluorescent plate reader (Molecular Devices, LLC, Sunnyvale, CA). Freshly prepared AAPH free-radical solution (140 mM in phosphate buffer) was added (50 μl), and the FITC fluorescence decay was monitored at 32-sec intervals for 40 min (excitation, 485 nm; emission, 538 nm; cutoff, 530 nm). ORAC values were calculated as the area under the curve using SOFTmax® Pro 5.2 Software (Molecular Devices, LLC, Sunnyvale, CA), and the data expressed in μmol Trolox equivalents.

### Statistical analysis

For the cytokine and immune cell measures, a non-parametric Wilcoxon/Kruskal-Wallis Rank sum test was performed on the differences in levels before and after supplementation. Illness and symptom data was analyzed by logistical regression with a generalized linear model and an exponential distribution. An over-dispersion parameter was used, due to larger-than-expected variance (JMP, version 8, Cary, NC). A nominal alpha level of 0.05 was used to declare statistical significance. A sub-group analysis of all primary indices was performed to determine and to address blinding issues, comparing those who guessed correctly (n = 5) with those who did not.

## Results

**Figure 1:**
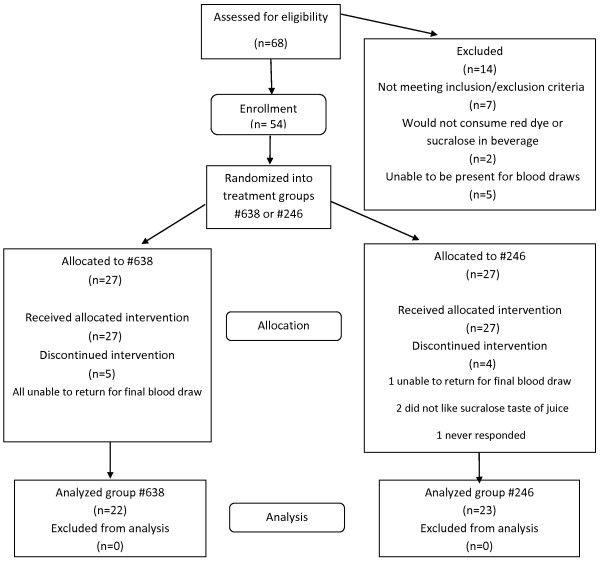
**Flow diagram: Study participant eligibility assessment, enrollment, group allocation and analysis.** People recruited for the study were assessed and those deemed eligible were enrolled. Subjects were randomized into one of two beverage groups. Bottles of the two beverages, cranberry and placebo, were received from Ocean Spray Cranberries, Inc. and labeled either #638 or #246. Subjects and investigators were blinded regarding the treatment groups. Statistical analysis was performed on data from all subjects completing the study. Investigators were unblinded (cranberry beverage: #638; placebo beverage: #246) following completion of the data analysis.

Of 68 people assessed for eligibility, 54 individuals enrolled in the study (Figure 1). During the study, 9 people withdrew: six because they were unable to return for the final blood draw, two because of the taste of the beverage, and one with no explanation. Of the 54 subjects enrolled, 45 (83%) completed the study. Overall compliance, determined by returned bottle count, was 96.1% ± 3.7% bottles of beverage (Table 2). All participants consumed at least 80% of their allocated beverage; therefore, no one was excluded from data analysis based on noncompliance.

**Table 2:** Demographics and cold and influenza symptoms

**Characteristic**	**Placebo beverage**	**Cranberry beverage**	**P**
**Demographic**			
*n*	23	22	
Age (y) ± SD^a^	24.0 ± 3.3	24.9 ± 5.8	
Male	5	9	
Female	18	13	
BMI^b^, initial	21.5 ± 2.7	23.2 ± 4.0	0.103
Change in BMI (kg/m^2^) ± SD	-0.095 ± 0.52	-0.063 ± 0.37	0.810^c^
No. blinded/guessed correctly (%)	23/23 (100)	5/22 (23)	<0.001
Compliance^d^ (%) ± SD	96.3 ± 4.6	95.9 ± 3.7	NSD^e^
**Cold and Influenza Symptoms**			
*n*^f^	20	15	0.624
Total incidence^g^	31	21	0.282^h^
Total cold and influenza symptoms	354	297	0.031^h^
Incidence of intestinal distress	35	15	0.021^h^
Total days missed work/school	29	20	0.329^h^
Total times reported a decrease in activity	28	16	0.154^h^

^a^SD, standard deviation ^b^BMI, body mass index ^c^*p* value derived from *t*-test ^d^Based on bottle count ^e^NSD, not significantly different ^f^Number of people who reported symptoms ^g^Number of colds and cases of influenza reported. Some individuals had more than one illness during the 10 wk period ^h^P value derived from *z*-test of proportions.

Regarding blinding, all subjects (100%) in the placebo group thought they were drinking the PB, while only five of the 22 people in the experimental treatment group (23%) guessed they were drinking the CB (Table 2). As there was a significant difference between the proportion of people guessing correctly versus the proportion of people guessing incorrectly (*p* < 0.0001, Fisher exact test), with nearly everyone in the study believing they were on the placebo, bias toward treatment was not anticipated to influence outcome. A subgroup analysis indicated that there were no significant differences in any of the parameters, but one, when comparing who guessed correctly with who guessed incorrectly. Those in the group that guessed correctly had slightly higher MIP1α levels at baseline compared to 10 week, or compared to either time point of those who did not guess correctly. Interactions between guessing correctly and time were not significant. Since this was observed in the baseline value, before anyone started the study, the biological significance of this statistical difference is unknown.

The total incidence of colds and influenza were not statistically different between the two groups (Table 2). However, the proportion of the total number of symptoms was statistically lower in the CB drinking group (*p* = 0.031). Since only 11.1% of the subjects thought they were consuming the active treatment, a bias by what they thought they were drinking was not suspected. The report of intestinal distress as a symptom was statistically greater (*p* = 0.021) in the PB group, as compared to the CB group. No other symptom showed any statistical difference. However, because subjects in the placebo group reported a few more symptoms in each category, the total number of symptoms was proportionally greater in that group compared to the CB group, and total symptoms were statistically different between the groups.

The percentage of specifically labeled PE^+^-FITC^+^ cells in the lymphocyte population (FSC/SSC) was determined at the blood draws before and after consumption, in both freshly isolated PBMC and in PBMC that were cultured for six days. Proliferation of γδ-T cells was significantly improved after CB consumption (Table 3) compared to the placebo. NK cell proliferation did not achieve significance when treatments were compared. There was no effect of CB on αβ-T cells, B cells or monocytes. The antioxidant activity of the serum was determined in de-proteinated samples, but the groups were not statistically different (data not shown).

**Table 3:** **Immune cell proliferation**^**a**^**fold change**^**b**^**after cranberry beverage consumption**

**Cell type**	**Placebo beverage**	**Cranberry beverage**	**P**
γδ-T cell^c^	1.20 ± 0.26^f^	3.86 ± 0.50	<0.001
NK^d^ cell^e^	0.15 ± 0.12	0.33 ± 0.19	0.068
αβ-T cell^c^	-0.16 ± 0.09	-0.16 ± 0.08	0.602
Monocyte^e^	-0.11 ± 0.29	-0.30 ± 0.45	0.883
B cells^e^	0.81 ± 0.16	1.32 ± 0.40	0.235

^a^After isolation (Day 0), cells were placed into culture in RPMI-1640 with autologous serum and PHA-L, IL-2 and IL-15 for 6 days ^b^A ratio of cultured cell numbers (Day 6/Day 0) was used to calculate proliferation fold changes. Baseline fold change values were subtracted from values obtained at 10 wk ^c^Percentage of the CD3^+^ population determined by 2-color staining ^d^NK, natural killer ^e^Percentage of total cells ^f^Wilcoxon/Kruskal-Wallis rank sums test was used to determine significant differences. Values were calculated for each subject and the mean ± SEM determined.

Cytokine levels in the supernatant fluids of 24 h PBMC cultured with PHA-L were determined, resulting in an observation of variability among subjects. Therefore, the change between cytokine secretion at 10 wk and secretion at baseline was compared between the placebo and treatment groups (Table 4). The ability of PBMC to secrete interferon-γ (IFN-γ) was significantly increased after CB consumption (*p* = 0.041). Other measured cytokines were not statistically different.

**Table 4:** **Cytokines secreted by PBMC**^**a**^**during 24 h culture**^**b**^

**Cytokine**	**Placebo beverage**	**Cranberry beverage**	**P**^**c**^
IL-1β	-519.7 ± 115.5^d^	-187.8 ± 107.1	0.088
IFN-γ	-24.8 ± 33.4	148.1 ± 80.2	0.041
TNF-α	189.7 ± 155.9	367.2 ± 140.2	0.452
IL-17	16.8 ± 11.3	26.2 ± 16.7	0.973
IL-1α	-36.5 ± 20.0	-27.9 ± 29.7	0.496
MIP-1β	968.3 ± 533.1	1120.0 ± 470.3	0.395
IL-13	-3.1 ± 7.1	-11.5 ± 10.6	0.156

^a^PBMC, peripheral blood mononuclear cells ^b^On Day 0, PBMC were cultured in RPMI-1640 with autologous serum and PHA-L, IL-2 and IL-15 ^c^P values are two-tailed based on the Wilcoxon/Kruskai-Wallis Rank sum test ^d^Values reported are the mean changes ± SEM of the 10-wk data minus the baseline data.

## Discussion

The primary predefined outcome measure was a change in the ability of γδ-T cells derived from peripheral blood immune cells to proliferate in *ex vivo* culture. The γδ-T cells showed an improved ability to proliferate in culture with PHA-L, after the CB was consumed. The cranberry fraction powder was prepared from the juice of cranberries and one serving of the low calorie CB (450 ml) contained polyphenol levels comparable to a serving of cranberry juice cocktail (250 ml). The proanthocyanidin fraction contained both A-and B-type linkages. Cranberries are known to have high antioxidant activity, yet serum antioxidant activity was not different between the groups or from baseline to 10 wk (data not shown). Pharmacokinetic studies that measure immediate accumulation of polyphenols in blood show that the turnover is rapid; about 2-4 hours [23-25]. Thus, a lack of change in serum antioxidant activity is not surprising when measured in the serum of fasting subjects, as we did in this study.

We propose that γδ-T cell proliferation *ex vivo* is a surrogate marker for immune function. We have shown in previous studies that their proliferation can be modified by diet [15-17]. The issue of bioavailability must be addressed since many studies show that polyphenols do not accumulate in the blood, but are rapidly metabolized. Intestinal immune cells are able to interact with the contents of the lumen because of their location in the Peyer’s patches and the intra-epithelium. Migration of intestinal immune cells is well documented [26,27] and migration patterns have been shown to be influenced by diet [28,29]. Although data specifically regarding γδ-T cells is lacking, we hypothesize that we measure changes in blood immune cells because they migrate after they have interacted with luminal bioactive compounds in the intestine.

In addition, larger molecules, at times, are able to translocate across the intestine and find their way to cells in the lamina propria and the mesenteric lymph nodes. Bacterial toxins are found intact in the blood or lymph [30,31]. Proteins are absorbed intact [32,33] and procyanidin dimers are transferred to the serosal side of enterocytes in the isolated small intestine [34] or in a Caco-2 cell model [35]. Jutila’s group has shown that the proanthocyanidin content of herbs interacts with receptors on the γδ-T cell and primes that cell [36]. After priming, the cell is able to respond faster, and to a greater extent, than if it were not primed. Direct interaction of dietary components in the lumen, or translocation of dietary components into the lamina propria, could result in priming of immune cells. Finally, changes detected in systemic blood cells may also occur because of the fermentation of unabsorbed polyphenols in the large intestine, resulting in bioactive compounds that are subsequently absorbed. The study was not designed to distinguish among these different mechanisms.

Changes in immune function may be responsible for a reduced number of cold and influenza symptoms. Although the incidence of colds and influenza were similar between the two groups, the total number of symptoms was lower after consuming the CB. Combining ‘missed work’ and ‘lower ability to perform a normal daily routine’ resulted in a *p* value of 0.056. This study was not powered on cold and influenza symptoms; it does not achieve statistical significance, so the idea that cranberry consumption has an effect on a health outcome is only a suggestion. Furthermore, the data on cold and influenza symptoms were self-reported and physicians did not confirm the presence of cold or influenza pathogens. However, Macintyre and Pritchard demonstrated that self-reported symptoms, as well as symptom severity, was highly correlated with assessments made by physicians [37], therefore we feel confident that the symptoms reported by the subjects accurately reflect the symptoms they had.

We examined cytokines based on previous *in vitro* data (manuscript submitted) in which PBMC from human donors were incubated with various cranberry fractions. For evaluation in this study, we chose those cytokines that were secreted in response to the addition of the cranberry fractions. The results from participants in this study showed the response of the cells to *ex vivo* stimulation was extremely variable. Drawing firm conclusions about the role cranberry plays in cytokine production is premature, yet the data suggest that cranberry contributes to an anti-inflammatory effect. Cranberry proanthocyanidins have been suggested as being anti-inflammatory in *in vitro* studies [38,39] and in rabbits [7]. One human intervention study showed that the level of urinary IL-6 was reduced in pregnant women who consumed cranberry juice compared to a placebo [8], yet other cytokines measured in that study did not achieve statistical significance, perhaps due to the small *n* of each group.

## Conclusions

In summary, daily consumption of a cranberry beverage containing cranberry polyphenols and proanthocyanidins at levels similar to commercially available juice for 10 wk, was effective in increasing the proliferation response of γδ-T cells and perhaps NK cells. Overall, the improved proliferation response was coupled with lower production of an inflammatory cytokine. Improvement of functional immunity, particularly of a cell located in the epithelium and responsible for barrier protection, might be another mechanism by which cranberry is able to maintain urinary tract health. These physiological changes could be, in part, responsible for our beneficial health outcome, a reduction of the number of illness symptoms.

## Abbreviations

AAPH: 2,2′azobis 2-amidinopropane dihydrochloride; CB: Cranberry beverage; ELISA: Enzyme-linked immuosorbent assay; FITC: Fluorescein isothiocyanate; NC: Not calculated; NK: Natural killer; NSD: Not significantly different; ORAC: Oxygen radical absorbance capacity; OSC: Ocean Spray Cranberries, Inc.; PB: Placebo beverage; PBMC: Peripheral blood mononuclear cell; PHA-L: Phytohemagglutinin; PE: Phycoerythrin; TCR: T-cell receptor.

## Competing interests

CK is an employee of OSC. SSP receives no other compensation from OSC outside of the funding for this research. The other authors have no conflicts of interest.

## Authors’ contributions

The study was conceived by SSP and CK, and SSP was responsible for the study design. MPN, CAR, CM, and RC contributed to the design of the study and were responsible for coordinating the intervention, performing the assays and writing some of the paper. JC helped with the statistical analysis. CK was responsible for the preparation, analysis and randomization of the beverages. SSP interpreted the data, wrote the paper, and is responsible for final content of the manuscript. All authors read and approved the final manuscript.
